# Bio-Inspired Approaches to Safety and Security in IoT-Enabled Cyber-Physical Systems

**DOI:** 10.3390/s20030844

**Published:** 2020-02-05

**Authors:** Anju P. Johnson, Hussain Al-Aqrabi, Richard Hill

**Affiliations:** 1 Department of Engineering and Technology, Centre for Planning, Autonomy and Representation of Knowledge (PARK), School of Computing and Engineering, University of Huddersfield, Queensgate, Huddersfield HD1 3DH, UK; 2 Department of Computer Science, Centre for Industrial Analytics (CIndA), School of Computing and Engineering, University of Huddersfield, Queensgate, Huddersfield HD1 3DH, UK; H.Al-Aqrabi@hud.ac.uk (H.A.-A.); R.Hill@hud.ac.uk (R.H.)

**Keywords:** security, internet of things, cyber-physical systems, hardware trojan horse, design for trust, field programmable gate qrray, bio-inspired engineering, spiking neural networks, astrocytes

## Abstract

Internet of Things (IoT) and Cyber-Physical Systems (CPS) have profoundly influenced the way individuals and enterprises interact with the world. Although attacks on IoT devices are becoming more commonplace, security metrics often focus on software, network, and cloud security. For CPS systems employed in IoT applications, the implementation of hardware security is crucial. The identity of electronic circuits measured in terms of device parameters serves as a fingerprint. Estimating the parameters of this fingerprint assists the identification and prevention of Trojan attacks in a CPS. We demonstrate a bio-inspired approach for hardware Trojan detection using unsupervised learning methods. The bio-inspired principles of pattern identification use a *Spiking Neural Network (SNN)*, and *glial cells* form the basis of this work. When hardware device parameters are in an acceptable range, the design produces a stable firing pattern. When unbalanced, the firing rate reduces to zero, indicating the presence of a Trojan. This network is tunable to accommodate natural variations in device parameters and to avoid false triggering of Trojan alerts. The tolerance is tuned using bio-inspired principles for various security requirements, such as forming high-alert systems for safety-critical missions. The Trojan detection circuit is resilient to a range of faults and attacks, both intentional and unintentional. Also, we devise a design-for-trust architecture by developing a bio-inspired device-locking mechanism. The proposed architecture is implemented on a Xilinx Artix-7 Field Programmable Gate Array (FPGA) device. Results demonstrate the suitability of the proposal for resource-constrained environments with minimal hardware and power dissipation profiles. The design is tested with a wide range of device parameters to demonstrate the effectiveness of Trojan detection. This work serves as a new approach to enable secure CPSs and to employ bio-inspired unsupervised machine intelligence.

## 1. Introduction

The adoption of sensors and embedded devices in cloud computing and the Internet of Things (IoT) requires systems with enhanced trust and security within applications [1,2,3]. The majority of high-value/high-profit businesses use and benefit from Internet-based computing, which relies on a large amount of data being collected and made accessible by connecting objects to IoT systems [4,5]. Within the IoT field, there is a rapid expansion in the area of radio frequency identification (RFID), sensors, and communications technologies, and their combined effect is to generate intellectual property (IP) [6], although there is less emphasis on the *protection* of IP. Cloud-based systems are a vital technology in this respect as they provide usefulness and accessibility to utility computing in terms of universal availability and timely access. However, to enhance trust and security, cloud computing needs more secure software and hardware solutions [7,8] to mitigate the risks of transporting IP-rich data outside of organisational networks and firewalls.

Technological advancements in the field of IoT are enabling the development of innovative products and services that rely on novel hardware platforms, forming Cyber-Physical Systems (CPS). One such significant improvement is the use of Field Programmable Gate Arrays (FPGAs) in cloud computing services and architectures [9]. Due to the high demand for computing resources, cloud and data centre architectures are moving towards hardware-accelerated computing. Recent studies show that FPGAs can outperform Graphical Processing Units (GPUs) [10] and, as a consequence, are increasingly being used in data centres [11]. The ability to reconfigure FPGAs makes them extremely powerful, as designs can be easily modified and updated once in service. Rather than the years required to build an Application Specific Integrated Circuit (ASIC) [12], design changes in FPGAs require only a few months. However, FPGAs are not completely immune to hardware and software vulnerabilities. Due to advancements in threat vectors, hardware vulnerabilities require further investigation [13]. Recent research shows an increase in the number of attacks upon hardware, indicating that there is a need for new hardware security primitives and Design for Trust (DFT) in hardware platforms [14,15].

Hardware Trojan Horses (HTHs) are manipulations of hardware Integrated Circuits (ICs) that weaken the security of a system. There are two essential characteristics of an HTH [16]. First, the HTH has a malicious intention, such as altering the device functionality, exposing sensitive information, or reducing circuit reliability. Second, the HTH is added to the device intentionally. The term intentional implies extra effort in the detection of the HTH, as they are developed especially to bypass traditional testing techniques. A Trusted Integrated Circuit (TIC) is an electronic circuit that is designed and developed to enhance trust in areas including IC design, manufacture, IP protection, and chip authentication [17]. To guarantee an HTH-free chip requires a demanding testing method. Two main classifications of HTH detection methods are (1) invasive methods and (2) noninvasive methods [18]. In the invasive approach, the manufactured IC is tested using invasive and destructive probing, which leads to either partially damaged or completely destroyed integrated circuits (ICs). However, the applicability of such approaches is minimal as the attacker is most likely to modify only a small random sample of chips in the production chain. The Trojan detection in the modified chips relies heavily on the probability of selecting the chip with the inserted HTH. Moreover, the method is expensive for the time and cost taken to test a single IC.

Logic-testing-based techniques and side-channel analysis-based techniques are two broad classes of noninvasive HTH detection methods. Logic-testing-based methods attempt to determine a deviation in functionality at various nodes of the circuit. As there is a vast taxonomy of Trojans in the domain, an adversary can exploit any one of them, needing a one-to-one correspondence between testing methods and the type of Trojan. Thus, a generic logic-testing method cannot be employed. Side-channel analysis is a broad approach where the techniques rely on a fingerprint of the IC, which is a measure of a physical quantity such as the supply current or path delays. IC authentication using Physical Unclonable Functions (PUFs) generally relies on device-parameters for security [19,20,21]. They work well for a variety of Trojans and IC designs with a range of complexities. Conventional approaches to side-channel-based methods are vulnerable to process variations, which can lead to failure in distinguishing between an HTH infection and a fault-free IC [22]. This work proposes an HTH detection method using bio-inspired principles that relies on the device parameters. The HTH detection unit is capable of considering natural process-dependent variations, thereby avoiding a false Trojan alert.

Investigations into how the human body responds to malignant growths have led to the development of an approach to identify the presence of Trojans in an electronic circuit. The human immune system is designed to recognize the cells that make up our bodies and to repel any foreign invaders such as viruses. An immune system adjusts with some level of variation, but higher levels of variation are displayed as diseases. We use a similar approach where a predefined device parameter variation is permitted to occur, yet when an IC parameter variation appears beyond a tolerance level, this is exposed using a reliable HTH detection unit. Our work uses unsupervised machine learning methodologies in a Spiking Neural Network (SNN) design. The design of the HTH detection process considers the influences of brain cells, including Astrocytes and gamma-aminobutyric acid (GABA)-ergic neurons. Astrocytes are glial cells in the central nervous system (CNS), play diverse roles, and are essential for a variety of critical neural functions [23]. GABAergic neurons produce gamma-aminobutyric acid (GABA), a neurotransmitter, which has critical roles in transforming the synaptic regulations in the brain [24]. SNNs have gained considerable popularity in embedded applications as they bridge the gap between machine learning (ML) and neuroscience. As these models are computationally complex for software implementations, they are not widely in use at present. However, due to advancements in embedded systems, they have become more accessible and have been the subject of research such as Brainscales [25], SpiNNaker [26], IBM True North [27], and Loihi [28]. Such research considers theoretical applications but is seldom applied to practical tasks and is not widely deployed. Recently, approaches to incorporating bio-inspired principles in enhancing security have attracted significant interest. In [29], the authors discuss SNN-based trojan analysis to explore the vulnerabilities of Denial-of-Service (DoS) attacks. Additionally, researches in side-channel analysis and machine-learning-based pattern detection have gained increased interest [30,31].

We use a modified SNN by combining the activities of glial cells in the brain termed astrocyte. The main aim of the proposed work is to implement a bio-inspired hardware Trojan-detection methodology suitable for networked applications. Spiking neural networks are proven to be one of the best models to mimic the brain-inspired method. Additionally, incorporating brain cells such as astrocytes and GABA interneuron is not viable in other neural-network models. To the best of our knowledge, SNNs are data-driven and event-driven and are potentially an excellent candidate for designing bio-inspired systems. The Spiking Astrocyte Neural Network (SANN) is a modification of SNN, which considers the contributions of astrocytes. This work proposes the use of SANN in hardware Trojan detection within an electronic circuit. The HTH detection unit is fault resilient by astrocyte-mediated synaptic regulation. We use unsupervised machine learning to implement stable signing for the Design Under Test (DUT) if the device parameters are in a permissible range. Any deviations from this behavior lead to a reduction in neuron-firing response. All presynaptic neurons fetch different device parameters to the postsynaptic neuron. During the training phase, the circuit learns to achieve a constant firing activity with the available parameters. We use a steep transmission probability (PR) curve between the neurons, which can be adjusted to incorporate new device behaviour in the future. For example, some circuits might require variable power and use a variable PR curve for transmission regulation between the presynaptic and postsynaptic pair. The stable firing activity produced by permissible device variation acts promotes occurrences of the device working, whereas failure would disable circuit functionality. This arrangement is specifically for HTHs, which are activated by increasing or decreasing the device parameters, such as varying the power, increasing/decreasing the temperature, or laser-based attacks on the device. These variations would trigger a firing fault in the output neurons of the network. Variability is adjusted to prevent unnecessary failures or false Trojan detection by using a tunable transmission probability curve. For safety-critical applications, the tunability curve is set at an extremely low standard deviation.

The organization of this article is as follows. In Section 2, some preliminary research in the area of spiking neural networks is presented, which includes the role of astrocytes in selectively propagating spiking information in the multi-layer neural network. Section 3 describes a primary Trojan detection circuit, acknowledging the contribution of astrocytes in the regulation of neural transmitters in a brain-inspired system. Section 4 presents the complete Trojan detection circuitry for discovering a combination of variations in the design. Section 5 introduces a Design For Trust (DFT) design methodology for device locking/unlocking based on device parameters. In Section 6, we discuss the design methodology adopted and various design parameters used in our experiments. This section includes our experimental results, which establish the effectiveness of the proposed scheme by analyzing the variations in device parameters. Finally, conclusions and future work are discussed in Section 7.

## 2. Fundamental Research

### 2.1. Learning in Spiking Neural Networks

To generate a constant firing activity for the SANN, we adopt a learning algorithm. In this approach, Spike Timing Dependent Plasticity (STDP) [32,33] together with Bienenstock, Cooper, and Munro’s (BCM) learning rule [34,35] are combined to develop the BCM-STDP rule [36,37]. The time difference between presynaptic and postsynaptic spikes is used to adjust synaptic weights. Equation (1) describes this STDP-BCM formulation.

(1) $$\delta w\left(\Delta t\right)=\left\{\begin{array}{c} +A_{0}.exp\left(\frac{\Delta t}{\tau_{+}}\right),\;\;\;\;\Delta t\leq 0\\ -A_{0}.exp\left(-\frac{\Delta t}{\tau_{-}}\right),\;\;\Delta t>0 \end{array}\right.$$

where δw(Δt) is the weight update, Δt is the time difference between presynaptic and postsynaptic spike events, $A_{0}$ is the height of the STDP-learning window controlling the maximum levels of weight potentiation and depression, and $\tau_{+}$ and $\tau_{-}$ control the decay rate of weight updating. The BCM learning rule modulates the height of the STDP plasticity window as a function of the neuron’s actual firing rate, according to Equation (2).

(2) $$A_{0}=\frac{A}{1+exp^{a(f-f_{0})}}-A_{n}$$

where the actual and target firing rates of the postsynaptic neuron are *f* and $f_{0}$, respectively, *A* represents the maximum height of the plasticity window. $A_{n}$ is the maximum height of the plasticity window for depression. The parameter *a* is constant and controls the opening/closing speed of the plasticity window. The value of *a* is found experimentally to be 0.1. The updated weights combine to produce a current which is injected back to the postsynaptic neuron, thereby establishing a constant firing activity.

The “*m*th” synapse between presynaptic neuron “*i*” and postsynaptic neuron *j* ( $S_{ijm}$) generates a current based on the synaptic weights given by Equation (3).

(3) $$I_{inj_{ijm}}=\eta .\left(W_{ijm}+\delta w\left(\Delta t\right)\right)$$

where η is a constant that is used to modulate the synaptic weights based on the transmission probability PR and the flow of current to the neuron is regulated as described in Equation (4). Section 2.2 describes PR formulation considering astrocyte–GABA interactions.

(4) $$I_{ijm}\left(t\right)=\left\{\begin{array}{c} I_{{inj}_{ijm}},rand\leq PR\\ 0,\;\;otherwise \end{array}\right.$$

where $I_{{inj}_{ijm}}\left(t\right)$ is the amount of current generated at time *t* by the synapse $S_{ijm}$. “rand” is a random function used to model the probabilistic synapse. $I_{ijm}\left(t\right)$ is a current released by the synapse on a successful probabilistic event described as in Equation (4). The total current injected to a postsynaptic neuron (j) (on a successful pattern detection by GABA–astrocyte interaction) is given by Equation (5).

(5) $$I_{total_{j}}=\left\{\begin{array}{c} \sum_{i=1,m=1}^{N,k}I_{{inj}_{ijm}}\left(t\right),\;\;E=1\\ 0,\;\;otherwise \end{array}\right.$$

where “*N*” is the number of presynaptic neurons (also the width of a pattern) and “*k*” is the number of paths between a pair of presynaptic and postsynaptic neurons. Based on the input pattern, postsynaptic neuron “j” learns to achieve the required spike rate. Learning is achieved using STDP and BCM rules. As per Equations (1) and (2), if the output frequency slightly deviates from the required output frequency ($f_{o}$), the weights of synapses are updated by a certain amount. If the input frequency varies significantly from the permissible level, the output frequency $f_{o}$ drops to zero.

### 2.2. Spike Flow Regulation in the Spiking Neural Network

Spike flow is regulated by following a bio-inspired activity-dependent transmission regulation in the brain influenced by astrocytes and GABA interneurons. Studies [24] establish that a reduced transmission of spikes to the postsynaptic neuron terminal occurs at a reduced presynaptic neuron spike rate. Also, for higher presynaptic signaling, the effect is the converse of that at low transmission probability, i.e., at a higher input transmission rate, a higher transmission of spikes occurs at the synapse. However, as the input spike rate increases beyond a threshold, the transmission of spikes also reduces and falls to zero. The above three observations are due to interactions at the synapse following a complex biological process where chemicals such as inositol 1, 4, and 5-trisphosphate ($IP_{3}$); calcium; and glutamate play important roles [24].

**Figure 1:**
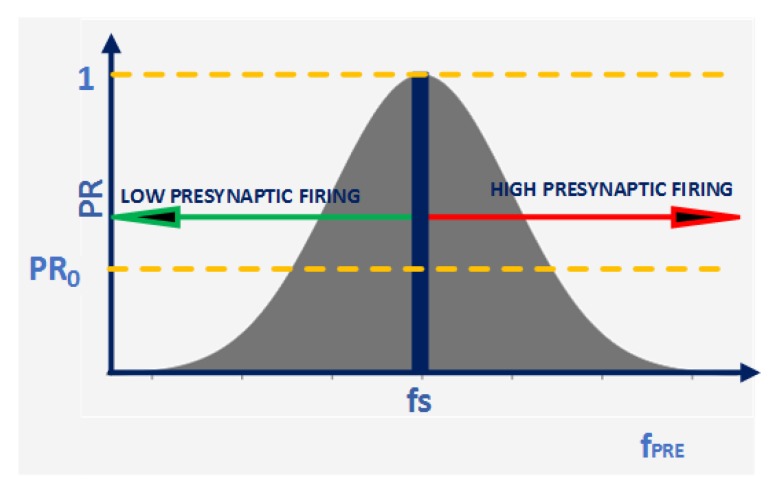
Activity-dependent transmission probability modulation in a Spiking Astrocyte Neural Network (SANN): A Gaussian distribution represents transmission probability of a tripartite synapse coupled to a Gamma-aminobutyric acid (GABA) interneuron. The Gaussian distribution parameters are adjusted to promote circuit variability and to avoid false triggering of the circuit. Critical applications use a narrow transmission probability curve.

In mathematical representation, the above three phenomena can be combined to represent a smooth neural transmission curve using a Gaussian distribution. The relation is modelled in Equation (6) and is represented in Figure 1. Note: Other functions such as triangular distributions and band-pass filters have also been experimented with, and they all provide identical responses.

(6) $$PR=exp^{(}\frac{{\left(f_{pre}-f_{s}\right)}^{2}}{2\sigma^{2}})$$

where $f_{pre}$ is the frequency of presynaptic neuron spikes, $f_{s}$ is the centre frequency of a pattern, and σ is the width of the Gaussian passband. Each interconnection between a pair of neurons is expected to fire at a rate close to $f_{s}$ at normal working conditions. Spikes are permitted to pass to the next layer of the network if the value of PR is within an acceptable range ($PR>PR_{0}$). This feature helps to selectively pass the information to the next layer if the elements are matched. The maximum value of PR is 1, which occurs when the presynaptic neuron fires at the same rate as the centre frequency.

## 3. Trojan Detection Unit Using SANN

**Figure 2:**
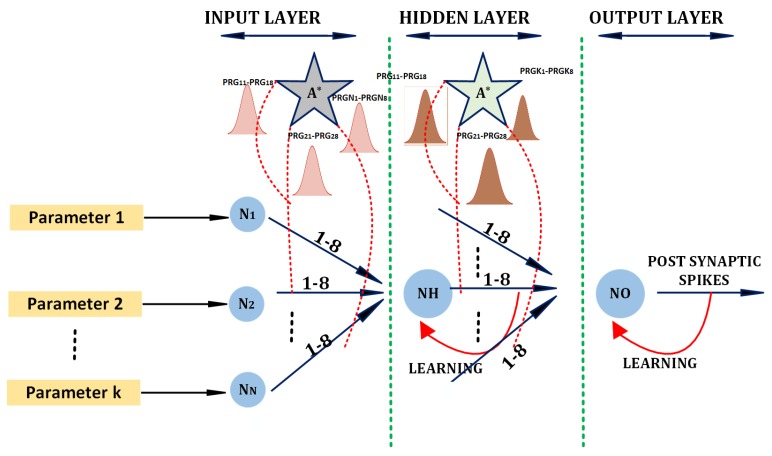
The basic unit for bio-inspired hardware Trojan detection using unsupervised learning methods Neurons $N_{1}$ to $N_{N}$ resides in the input layer of the network. A hidden layer contains $2^{N}$ neurons each, identifying a pattern. The neuron $N_{H}$ represents a hidden-layer neuron used for identifying a particular pattern of device parameters. A set of responses of the hidden-layer neurons combine to produce a stable firing activity in the output layer neurons. $N_{O}$ is an output layer neuron. Astrocytes reside between the layer for spike transmission regulation based on the patterns. There are *K* (8 in our experiments) parallel paths between each pair of neurons in adjacent layers. Based on the input pattern, the output neuron $N_{O}$ learns to achieve the required spike rate under a Trojan-free condition. The spike rate drops to zero if device parameters are altered beyond the permissible level, indicating the presence of malicious activity.

Figure 2 shows a multi-layered network (3 layers shown) with *N* neurons in the input layer. Each of the input layer neurons produces a constant firing activity for a given device parameter $D_{i}$. The device parameter is allowed to have a deviation of $D_{i}\pm \delta_{i}$ to incorporate natural device variability. The input neuron $NI_{i}$ produces spikes of frequency centered around $FI_{i}$ if the device parameters are in the permissible range. The spike frequency of $FI_{i}$ is directly proportional to the device parameter variations. If the device variations are not in the permissible range, $NI_{i}$ delivers a spike of a frequency widely deviating from $FI_{i}$. Similarly, based on the device parameters linked to each input neuron, spikes of predefined frequencies are generated.

All input layer neurons in the system that are working in a fault-free state are expected to produce spikes around frequency $FI_{i}$. Various patterns of device parameters can be formed using the input neuron responses. The input layer spike informs the second layer (hidden layer) of neurons. For example, pattern $P_{1}$ corresponds to the violation of all input device parameters (an equivalent of all zeros in binary). The $2^{N}$th pattern would point to all device parameters in the permissible level. Based on the amount of permissible variability, the number of patterns to be verified also increases. For a safety-critical system, we may require all device parameters to be in the permissible level. In this case, the only pattern to be checked is the $2^{N}$th pattern, and that represents all of the input neurons producing around $F_{1}$ frequencies. Some systems can have a permissible variation in one or more parameters. This scenario would lead to a pattern with a mixture of frequencies.

The number of neurons in the hidden layer equals the number of patterns formed with the input neuron frequencies. The hidden neuron layer corresponds to various patterns in the network. $NH_{j}$ would produce a frequency of $FH_{1}$ if all its presynaptic neurons produce frequencies in their respective ranges. Otherwise, $NH_{j}$ produces a frequency of $FH_{0}$. Thus, device parameter violations get projected in respective hidden-layer neuron responses. An astrocyte layer residing between the input and the hidden-layer neuron verifies and permits (or prevents) spike transmission using transmission probability regulation. The interactions of astrocytes with the GABA interneurons at the tripartite synapse influences transmission regulation, and details are provided in Section 2.2. Multiple parallel paths exist between any pair of neurons in the adjacent layer; parallel paths aid the building of postsynaptic potential and increase the security of the Trojan detection circuitry. Astrocytes permit a spike transmission across a layer if there exists at least one path with the required spike frequency between every pair of neurons.

The response of output layer neurons represents the nature of patterns observed. For example, a particular design might allow a different set of combinations of device variation. An increase or decrease in device current for a corresponding deviation in device voltage would be a permissible combination of device variation. Hence, one of the patterns would represent a particular current/voltage characteristic and a different hidden-layer neuron pattern would represent a different set of current–voltage components. Both of the above patterns are correct responses of the device. It follows that an output layer neuron would fire at a predetermined rate if any of these hidden-layer patterns were satisfied. Essentially, the three-layer network implements a binary expression as a Sum-Of-Product (SOP), where the hidden layer evaluates the product expression, and the output layer neuron evaluates the summative expression. There exists the same number of output layer astrocytes as the number of output layer neurons. The astrocytes verify if the pattern arriving in the output layer neurons forms the requirement. More complex pattern behavior can be created to produce a multi-layered network with more than three layers.

If the device parameters are at the permissible level, spikes in the system follow Equations (1) and (2) to update the synaptic weights. The updated weights combine to produce a current which is injected back into the postsynaptic neuron, thereby establishing a constant firing activity. When device parameters are not in the permissible level, some of the interconnections between the neurons produce incorrect spike frequencies. The astrocytes block any incorrect patterns, and hence, the firing activity drops to zero.

This design is tunable to support slight variability in the device parameters while, at the same time, reducing susceptibility to malicious intrusions. Spike flow is regulated by following a bio-inspired activity-dependent transmission regulation, as seen in the brain. Below, we briefly describe the bio-inspired principle.

The width of the Gaussian passband described in Equation (6) represents the amount of tolerance expected in device parameters. Various device parameters introduce different levels of deviation under normal operating conditions. Hence, the value of σ varies according to acceptable levels. Increasing the standard deviation leads to Trojans remaining undetected, and reducing the standard deviation may lead to false alerts. The value of σ is the designer’s choice based on the particular application. For critical applications, the Gaussian bandwidth is adjusted to follow a narrow passband to alert for any slight variation in the circuit.

## 4. Detailed Trojan Detection Unit for Large-Scale Cyber-Physical Systems

**Figure 3:**
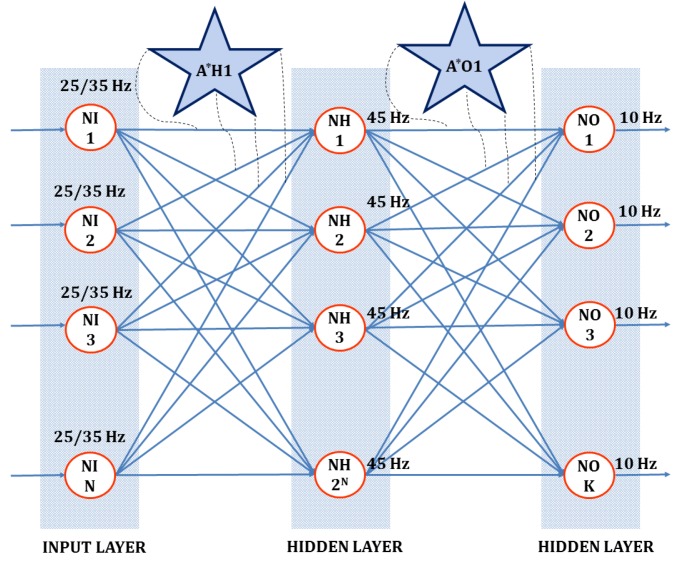
Three-layer spiking astrocyte neural network neurons $N_{1}$ to $N_{N}$: each receives synaptic input corresponding to the device parameters collected by various sensors. Based on the number of device parameters to be observed (*N* in our experiments), there exist *N* neurons in the input layer of the network. The number of neurons in the hidden layer correspond to the number of patterns that can be formed using the input neurons, leading to $2^{N}$ neurons in the hidden layer. The response of output layer neurons depends on the combined effect of the monitored device parameters. If the preferred combination of parameters in the network is *K*, then we have *K* neurons in the output layer of the network. There exist $2^{N}$ astrocytes between the input and hidden layer ($A^{*}H$), each guiding the flow of current to the hidden-layer neuron depending on transmission probability. Similarly, there exists a layer of astrocyte between the hidden layer and output layer counting to *K*, based on the parameter combination to be observed.

Figure 3 presents a fully connected three-layer spiking astrocyte neural network. The system generates a stable response with a balanced device parameter. The system consists of *N* input and *K* output neurons in the input and output layers of the network, respectively. The value of *N* is chosen based on the number of device parameters to be verified.

Based on the predefined device parameter levels, the input layer neurons fire at a specific rate. Variations in device parameters from the preset values lead to changes in the firing pattern of the input layer neurons. The second layer of the network contains $2^{N}$ of hidden-layer neurons, formed to identify all possible combinations of firing activity of the input layer neurons. There exists a layer of astrocyte to permit the flow between input and hidden-layer neurons by controlling the synaptic transmission.

Section 2 provides details of synaptic transmission regulation by astrocytes. Each hidden-layer neuron is associated with an astrocyte, and there are $2^{N}$ astrocytes between input and hidden-layer neurons. The number of hidden-layer neurons and astrocytes corresponds to the patterns in the input layer. This firing activity of input layer neurons and the associated response of the hidden-layer neurons generates current based on a combined BCM-spike-dependent plasticity rule. The hidden-layer neuron generates a stable firing rate if an observed pattern is correct based on the astrocyte transmission regulation. If the pattern is incorrect, the hidden-layer neuron does not produce any firing activity. The third layer of the network provides a combined response based on the activity of hidden-layer neurons connected to it. The astrocytes present between the hidden layer and the output layer control the flow between these two layers based on the correct patterns. A predefined stable firing activity of the output layer neuron corresponds to a Trojan-free circuit. The primary component of the proposed Trojan detection circuit is a neuron. The proposed design works with any spiking neuron representation; however, we encourage the use of the Leaky Integrate and Fire (LIF) neuron model [38]. The LIF neuron requires low computing resources and minimal tuning parameters for implementation, suiting compact hardware deployment. Similarly, all other components/modules used in the circuit implementation also have minimal hardware footprints to support lightweight applications and IoT hardware resources. The representation of a LIF neuron is presented in Equation (7).

(7) $$\tau_{mem}\frac{dv}{dt}=-v\left(t\right)+R_{mem}.I_{total}$$

where $\tau_{mem}$, *v*, $R_{mem}$, and $I_{total}$ are the time constant, membrane potential, membrane resistance, and current injected to the neuron, respectively. When the membrane potential reaches a threshold voltage, the membrane potential is brought back and held at 0V, following a nominal refractory period. We use the Euler method of integration to evaluate this expression.

A second important component is pattern identification by astrocytes. We use *k* parallel connections between pairs of neurons in two adjacent layers. The value of *k* is selected to provide sufficient self-security in the detection circuit. A higher value of *k* prevents the HTH detection unit from faults, either by a malicious intruder or by random faults in the circuit. Each parallel path implements the same flow of spikes between layers but with a predefined variable delay. Delay is introduced to promote the building of postsynaptic potential in spiking neurons. The higher the value of *k*, the more it leads to higher resource consumption in the circuit, and hence, a trade-off is required between the design size and security. Astrocytes permit the flow of spikes between input and hidden layers if they satisfy the relation modeled in Equation (8).

(8) $$E_{H}=\wedge_{j=1}^{N}\left(\vee_{i=1}^{k}\left(PR_{ji}\geq PR_{0}\right)\right)$$

where $E_{H}$ denotes the transmission of spikes to the next layer (1 = permit presynaptic spikes and 0 = disable presynaptic spikes). *N* is the number of neurons in the input layer, and *k* is the number of parallel paths between adjacent layers. $PR_{0}$ is the minimum transmission probability required to permit a spike through the parallel paths between the neurons. Once the pattern is detected ($E_{H}=1$), and spikes passed to the neuron NH, it learns to achieve a constant firing activity. Similarly, all hidden-layer neurons detect various combinations of input patterns. Identifying the response of various patterns in the hidden layer generates a combined response at the output layer. The astrocyte permits the flow of spikes between hidden and output layers if it satisfies the relation modeled in Equation (9).

(9) $$E_{O}=\wedge_{j=1}^{p}\left(\vee_{i=1}^{k}\left(PR_{ji}\geq PR_{0}\right)\right)$$

where $E_{O}$ denotes the transmission of spikes to the output layer (1 = permit presynaptic spikes and 0 = disable presynaptic spikes), *p* is the number of hidden-layer patterns (neurons) to be combined to generate the response, and *k* is the number of parallel paths between a pair of presynaptic and postsynaptic neurons between the input and hidden layers. $PR_{G0}$ is the minimum transmission probability required to permit a spike through the parallel paths between the neurons. Once the pattern is detected ($E_{O}=1$) and spikes are fed back to the neuron NH, and it learns to achieve a constant firing activity. If the output layer neurons fail to produce a predefined stable activity, this indicates the presence of variability in device response and is likely to be induced by malicious activity. Section 2.2 defines how to choose the transmission probability curve to avoid false Trojan alerts in the device.

## 5. Design for Trust

**Figure 4:**
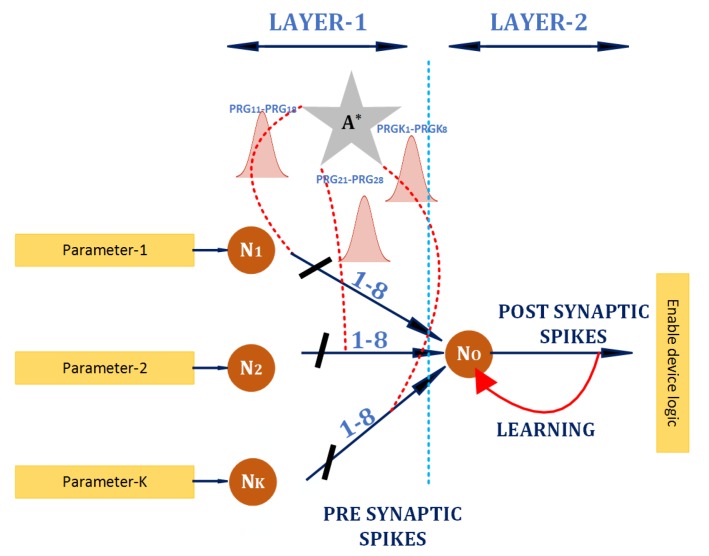
SANN-Design for Trust (DFT) logic-locking unit: Input layer consists of neurons representing different device parameters. The second layer neuron learns the pattern and produces a stable firing activity, which is used to derive an enabling logic for the design. Any considerable deviation from the pattern leads to ceasing of firing activity, and the device is locked.

The proposed architecture of Design for Trust (DFT) should be incorporated with the general CPS system in order to ensure real-time detection of a Trojan. A pattern in any area of the CPS is used to lock the respective part of the device logic by adjusting the learning curve to the permissible range. The basic DFT subblock implemented on an FPGA is with firing activity 54 for parameter 1, with firing activity 64 for parameter 2, and with firing activity 74 for parameter 3. A time window of 1024 clock cycle sets calculates the firing activity. Figure 4 represents the SANN-DFT logic unit for establishing device locking. The biological processes involved in a SANN system requires a high amount of hardware consumption for its precise representation. Hence, we approximate the fundamental equations to generate a compact hardware architecture. A moving average is used to determine the spike frequency of neurons with a window of size of 1024 clock cycles. Here, we use neurons in layer-1 to produce spikes corresponding to the side channel parameters. The neurons are implemented using the LIF equation, where the input current relates to the device parameter.

The second layer contains only a single neuron, which detects the presence of a unique pattern. This neuron is designed to fire at a rate of 100 spikes in a window if the pattern identification is successful. Hence, this structure requires only two layers with a single neuron in the second layer. Based on the configured parameter, the design produces a stable firing activity at the output of the neuron in layer-2. A stable firing activity is chosen as an enable/disable signal for device locking. Due to the stabilising nature of the design, minute variations of device parameters are filtered and do not produce any unreliable activity and unnecessary locking of the device.

In this work, we consider minute variations as typical behavior of the design, as they mostly cannot cause any malicious activity. Any considerable deviation in the device pattern triggers a low-firing activity instantly. Neurons in layer 1 facilitate the transmission of spike trains to the next layer. There are 8 parallel variable delay paths between every pair of presynaptic and postsynaptic neurons. Parallel paths allow the postsynaptic potential to build up neurons in layer-2. Additionally, they minimize any chance of attacks by circuit modifications in the parallel paths of the SANN-DFT logic-locking unit. An attacker needs to modify all of the eight connections in the unit to break the device-locking scheme. A fault in all eight connections would lead to a complete shut down of the logic area.

Following an initialization time, stable firing activity crossing 80% the target frequency enables the working of the Design Under Testing (DUT). The enable logic guarantees the recognition of the pattern. Any modifications of the parameters lead to the design being disabled. The eight parallel paths have different synaptic weights, and hence, controllability and observability are different in these paths. Hence, this would also prevent attacks targeted at the SNN-DFT logic-locking unit. Neurons in layer-2 produce a constant activity based on the learning mechanism. Nodes with low controllability and low visibility are targets for Hardware Trojan insertion, and inserting an SNN-DFT logic-locking unit at these nodes would reduce the total number of SNN-DFT logic-locking units in the design.

The unsupervised learning methodology quickly recovers any malicious or naturally occurring faults in one or more synapses by updating the weights in the healthy synaptic pathways. This recovery increases the reliability of the scheme. Any spike rate falling below 70% of the targeting rate locks the device from usage.

## 6. Experimental Results

### 6.1. Experimental Setup

The proposed architecture of the Trojan detection circuit is implemented in Xilinx Vivado 2018.1 and ISE 14.7. Euler integration evaluates the LIF neuron expression with a fixed time step of *t* = $2^{10}$ clock cycle. For hardware implementation, Equation (2) is approximated using a straight line and Equation (1) is approximated by powers of 2 (shift operations). A rectangular band-pass filter approximates the Gaussian filter representing the transmission probability in Equation (6). The system is benchmarked against a fault-free Trojan detection SANN unit implemented on the FPGA. We deliberately introduced changes in the device parameters to mimic the presence of Trojans to demonstrate the device-locking phenomenon. In the permissible range of device variations, the design produces a stable enable signal to drive the design. The proposed system could successfully establish the device-locking phenomenon if the side channel parameters vary above the permissible limit. Table 1 provides design parameters for a SANN-Design for Trust (DFT) unit implemented in this work considering of three variable device parameters. Firing rates of the neurons implemented by an FPGA-based approximation of a SANN system is compared with a Matlab-based software implementation. Results illustrate that the FPGA-based approximation produces results comparable with the simulation results.

### 6.2. Hardware Results on Xilinx Artix-7 FPGA

The proposed Trojan detection circuitry is implemented on the Xilinx Artix-7 FPGA board. The device-locking circuitry is implemented on an FPGA and is monitored using a *Integrated Logic Analyzer* (ILA). Power estimation of the circuits was carried out using *Xilinx Power Estimation and Analysis Tools*, and delay estimation was carried out using *Timing Closure and Design Analysis*. Table 2 reports the hardware resource footprint of the proposed models. Estimated total on-chip power is also presented in Table 2. The hardware utilization increases with the number of synapses, which operates based on a BCM-STDP rule. We use 32-bit operations for determining the synaptic weights. We are working towards a Spike Driven Synaptic Plasticity (SDSP) [39]-based synaptic rule, which would drastically reduce the synaptic weight as they perform 1-bit operations. Another operation that consumes hardware is the calculation of transmission probability (Equation (6)). This equation replaces linear approximations, which would further minimize the hardware overhead.

The proposed fault-tolerant learning mechanism in a SANN can be incorporated with reduced hardware overhead and power consumption, establishing its usability in resource-constrained applications. The proposed system implemented on an FPGA achieves an acceleration of $10^{4}$ compared to the software simulation (Matlab), which indicates the viability of this approach for real-time cryptographic applications. In biological systems, independent units perform computation in parallel. For real-world applications, this parallelism can be exploited to execute tasks orders of magnitude faster than in software. One aspect of the proposed model is that it operates at an accelerated biological timescale; similar concepts are illustrated in our previous works [40,41].

### 6.3. SANN-DFT Initialization

**Figure 5:**
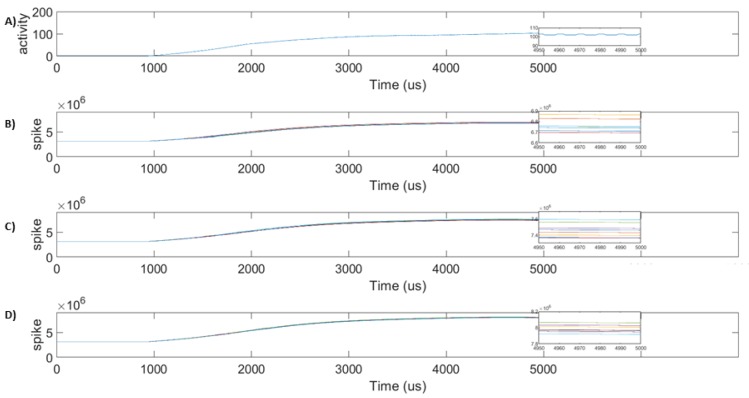
Synaptic weight updates during initialization: (**A**) Firing rate of $N_{o}$ in of SANN-DFT unit during the initialization phase. All synapses between neurons in layer one and layer two are healthy, enabling eight parallel paths of variable delays to contribute to the firing activity. A stable firing rate of 100 spikes/1024 clock cycles is established around 50 us. (**B**) Synaptic weights between $N_{1}$ and $N_{o}$ are shown. $N_{1}$ has a spike rate of 54 spike/1024 clock cycles. The value is zoomed around 50 us to show that different synapses update at different rates. (**C**) Synaptic weights between $N_{2}$ and $N_{o}$ are shown. $N_{2}$ has a spike rate of 64 spike/1024 clock cycles. (**D**) Synaptic weights between $N_{3}$ and $N_{o}$ are shown. $N_{3}$ has a spike rate of 74 spike/1024 clock cycles. Synaptic weights are different because of the variable delayed paths in the network and the relation between input and output spikes as per the BCM-STDP learning rule.

Three input spike trains of frequencies 54 spikes/window, 64 spikes/window, and 74 spikes/window are used for testing the basic functionality of the SANN-DFT unit. The above spike rates correspond to the parameter values of 20 mA, 22 mA, and 26 mA at three locations of the DUT. These input spike trains are compatible with the center frequency of the associated synapses, and hence, the astrocyte permits the spike to pass to the neuron $N_{o}$. The target frequency of neuron $N_{o}$ is set to be 100 spikes/window. A standard learning phase will occur, and the spike rate gradually increases during the training phase and eventually stabilizes at the target frequency of 100 spikes/window in 0.5 μs, as shown in Figure 5A. Figure 5B–D shows the synaptic weights, which show a slow rise over the learning period and stabilize at 50 us. Additionally, the system is tested with presynaptic spike train frequencies varying from center frequency and in the permissible range. No noticeable change is observed in the firing activity (results not shown), proving that the design is stable against natural variations.

### 6.4. Hardware Trojan Detection

The effects of HTH attacks on various platforms have been widely explored. Hardware Trojan insertions range from cryptographic hardware [42,43] to remote dynamic configurations on reconfigurable IoT platforms [44,45]. We assume that a high-frequency clock signal is used to drive the Trojan detection unit, which can be generated by using dedicated clock generators such as the Mixed-Mode Clock Manager (MMCM) module [46].

We apply the proposed SANN-based hardware Trojan detection method to the designed hardware Trojans reported in References [43,44,45] as they are relevant to reconfigurable platforms. A characteristic feature of the above research is the method of launching the attack from a remote location by dynamically altering the clock of the design. Applying the proposed Trojan detection circuit requires parameters to be defined. One setting which exposes the Trojan of this nature is the measure of clock width of the design. A high-frequency clock can be used to measure the width of the design clock. As the Trojan detection unit runs faster than the design clock, the design would be halted/disabled, following a failed pattern recognition. Also, other factors, such as a power or voltage profile, can be used as a parameter in the design.

**Figure 6:**
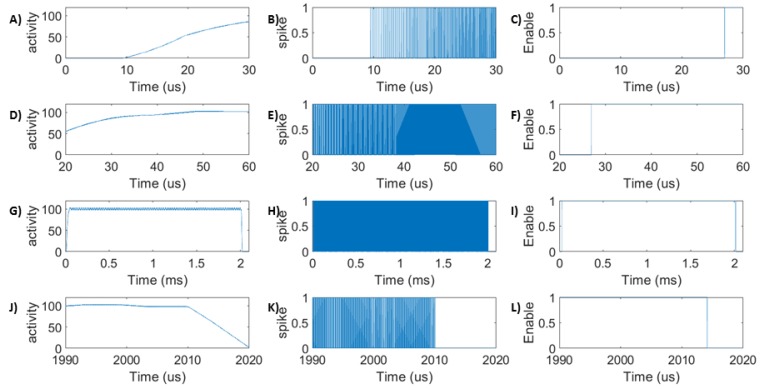
SANN-DFT Trojan detection: The activities of output layer neurons at various time slots: (**A**) the initial stage of output neuron activity which rises gradually from 0; (**D**) an intermediate activity rise stage; (**G**) the desired rate of 100 spikes is achieved in this time slot. The desired rate is achieved around 50.0 us. A Trojan is inserted at 2 ms, which changes the device parameters; and (**J**) the activity following the Trojan insertion decays to zero. The activity fully decays by 20 us after Trojan insertion. The spike products by output layer neuron: (**B**,**E**) during the initialisation phase, the spike rate gradually increases and stabilises (**H**) when the desired rate is achieved. (**K**) After Trojan insertion, the spike gradually ceases. The signal required to enable the working of DUT: The SANN-DFT unit enables the DUT to work only if the activity is above 80 spikes in a window. (**C**) The activity is too low to trigger the enable signal; (**F**) the activity needs to further increase to enable the DUF; (**I**) the spike activity is greater than 80 in this time slot, which enables the DUT; and (**L**) after Trojan insertion, activity drops below 70, which disables the DUT. The enable signal requires 14 us after Trojan insertion to fall to zero.

Figure 6 corresponds to a set of activities occurring in the SANN-DFT unit following a Trojan insertion. The DUT is enabled to perform its functionality only following an initialization stage of the SANN-DFT unit. The initialisation stage corresponds to the time taken by the SANN-DFT unit to achieve at least 80% of the targeted output neuron activity. The unit is designed to achieve 100 (Any desired value can be chosen.) spikes in a window. A spike rate of around 80 spikes in a window enables the working of design under test (DUT). Any spike rate below this value triggers a halt. The design takes around 50 us for initialization. At 2 ms, a Trojan is inserted, which changes one of the parameters outside the permissible range. This output firing activity degrades thereafter. More details are presented in Figure 6.

### 6.5. Fault Attack on Trojan Detection Unit

**Figure 7:**
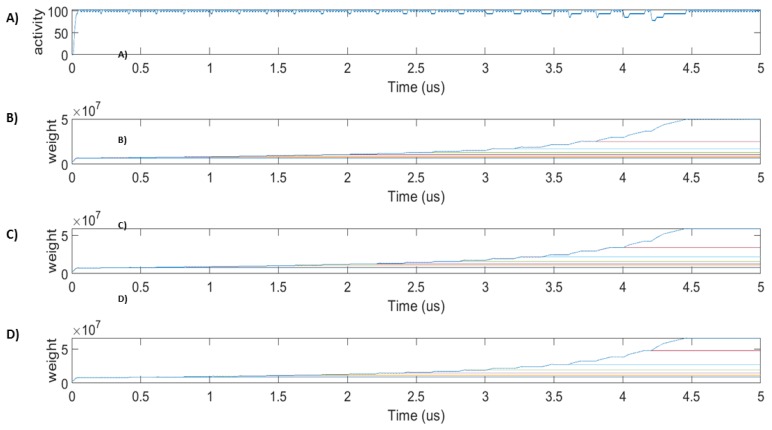
Firing rate of $N_{o}$ and synaptic weights under different faulty conditions: (**A**) Synapses between neurons in layer-1 and layer-2 induced faults gradually. A stable firing rate of 100 spikes/window is established in all faulty cases. (**B**) Synaptic weights between $N_{1}$ and $N_{o}$ under faulty synapses: We induce gradual faults on the system after every 200 μs. After every fault injection, the weights of healthy synapses update to a higher value to regain the target firing activity. Only one synapse of $N_{o}$ is left unbroken after 4200 μs. (**C**) Synaptic weights between $N_{2}$ and $N_{o}$ under faults of different percentages. (**D**) Synaptic weights between $N_{3}$ and $N_{o}$ under faults of different percentages. We can see that, for each fault percentage, the systems relearn the synaptic weights for establishing a constant firing rate similar to the fault-free results. The broken synapses do not increase weight.

To evaluate the fault-resilient nature of the proposed SANN Trojan detection unit, the spike train frequencies of N1, N2, and N3 were set to the center frequency of the Gaussian PR curve (54, 64, and 74 spikes/1024 clock cycles), and the SANN was trained with a target frequency for $N_{o}$ of 100 spikes/window. Faults are purposefully injected into the system gradually after every 200 μs. For example, at 200 μs, one of the synapses between $N_{1}$ and $N_{o}$ is broken; we can see an activity drop in Figure 7A, which subsequently causes the learning window to reopen and the learning process to restart. Likewise, we gradually increase the faults in synapses of all presynaptic neurons of $N_{o}$. At 4200 μs, 7 synapses between all presynaptic neurons and $N_{o}$ are broken. One synapse is left fault-free because learning does not happen without a healthy connection between a pair of neurons. The modulated weights of synapses associated with $N_{1}$, $N_{2}$, and $N_{3}$ are shown in Figure 7B–D. In all cases of faults, the firing rate is above 70 spikes/1024 clock cycles, providing a stable enable signal for the DUT. It is evident that the fault repair happens at a rate of μs, proving to be a very efficient scheme for practical cases. This proves the reliability of the HTH detection unit against malicious fault insertions.

## 7. Conclusions

In this article, we discussed how the bio-inspired approach of anomaly detection could be used in a reconfigurable platform for real-time Trojan detection. Unsupervised machine learning is used in the spiking neural network-based design for analyzing patterns at various locations of the reconfigurable platforms employed in CPS. The design grows in size, with the number of parameters to be analyzed. We recommend employing smaller units of the design at various/critical locations to monitor any undesired behavior. Since the circuit works extremely fast (microseconds), it indicates its suitability for real-time cryptographic Trojan analysis platforms.

The concept is derived by incorporating various bio-inspired principles, particularly the activities of GABA–astrocyte interactions in the selective transmission of pieces of information across a multi-layer network. The ability of the brain to achieve homeostasis is utilized to provide minute variations in the design, which are natural and not a threat. The SANN-DFT logic-locking unit is adaptive to minute changes in the system and does not trigger unnecessary device locking. Even if some of the device parameters vary, the system produces a stable “device enable” signal in real-time, which has no noticeable variation according to the parameter changes. Also, multiple paths are supported in each layer of the networks to avoid malicious circuit alterations in the design, which further increases the security of the Trojan detection unit from malicious modifications.

First, our work is implementable with reduced hardware resources, power dissipation, and propagation delay, leading to a scalable solution for reconfigurable deep-layer neural network architectures. Second, the proposed idea is demonstrated on an FPGA system that achieves real-time computation ($10^{4}$ times faster than the biological timescale (1 ms)). One reason is its ability to work in an accelerated biological timescale. The system can effectively establish a stable Trojan detection functionality with a minimum of 1 interconnection (healthy synapse) between a pair of presynaptic and postsynaptic neurons.

Future work shall investigate bio-inspired FPGA-based SANN designs for automotive embedded systems to address a variety of concerns including security, performance, fault-tolerance, reliability, and scalability. We mainly target applications of the proposed FPGA-based SANN systems in safety-critical CPS/robotic missions for implementing a real-time responsive system establishing satisfactory fault resilience. Emerging technologies such as micro-electromechanical systems (MEMS) are a promising solution for future implementations. MEMS configurations using nanoelectromechanical (NEM) [47] designs enhance the deployment of the FPGA-based system directly on the application site, closer to sensors and actuators, and eliminate heat protection circuity in the designs as they can work up to $225^{\circ }$C. Additionally, this deployment eliminates the latency associated with interfacing FPGA systems to on-site robotic controllers.

Our work considers different concepts in a spiking neural network to design a reliable FPGA-based Trojan detection platform, and the proposed design is appropriate for FPGA-based applications such as in clouds, IoT, and CPSs, where security is a critical factor. This research work constitutes a vital step in biologically inspired security for hardware applications.

## Figures and Tables

**Table 1:** Design parameters for SANN-DFT Unit.

Parameter	Parameter Description	Value
LIF * Neuron		
**$V_{thi}$**	Threshold voltage of input layer neurons	15 mV
**$V_{th}$**	Threshold voltage of hidden/output layer neuron	68 mV
**$P_{1}$**	Device parameter 1	20 mA
**$P_{2}$**	Device parameter 2	22 mA
**$P_{3}$**	Device parameter 3	26 mA
**$R_{mem}$**	Membrane resistance (all neurons)	1MΩ
**$\tau_{mem}$**	Membrane time constant (all neurons)	10 ms
BCM ** Learning Rule: approximated using linear equation		
***A***	Maximum height of plasticity window for excitation	1
**$A_{n}$**	Maximum height of plasticity window for depression	0.5
**$a_{0}$**	A constant	0.1
**$F_{s}$**	Target firing activity of hidden/output layer neuron	100/1024clocks
**Transmission probability: approximated using a band-pass filter**		
**σ**	Standard deviation	10
**$f_{s_{1}}$**	Centre frequency of pattern 1	54/1024clocks
**$f_{s_{2}}$**	Centre frequency of pattern 2	64/1024clocks
**$f_{s_{3}}$**	Centre frequency of pattern 3	74/1024clocks
**Synaptic weights**		
**$w_{0}$**	Initialized value	48
**η**	A scaling constant	0.0625

Note: * LIF Neuron: Leaky Integrate and Fire Neuron; ** BCM Learning Rule: Bienenstock–Cooper–Munro Learning Rule.

**Table 2:** Implementation overhead of the SANN-DFT presented in Figure 4.

Parameter/Components	Slice	Slice Reg	LUT	DSP
**Hardware Consumption**	14,471	33,707	25,065	30
**Total On-Chip Power**	0.082W			
